# Effector Memory T cells Are Associated With Atherosclerosis in Humans and Animal Models

**DOI:** 10.1161/JAHA.111.000125

**Published:** 2012-02-20

**Authors:** Enrico Ammirati, Domenico Cianflone, Viviana Vecchio, Michela Banfi, Anna C. Vermi, Monica De Metrio, Liliana Grigore, Fabio Pellegatta, Angela Pirillo, Katia Garlaschelli, Angelo A. Manfredi, Alberico L. Catapano, Attilio Maseri, Alessio G. Palini, Giuseppe D. Norata

**Affiliations:** Clinical Cardiovascular Biology Centre, San Raffaele Scientific Institute and the Università Vita-Salute San Raffaele, Milan, Italy (E.A., D.C., M.B.); Flow cytometry Resource Analytical Cytology Technical Applications Laboratory, San Raffaele Scientific Institute and the Università Vita-Salute San Raffaele, Milan, Italy (V.V., A.G.P.); Clinical Immunology Unit, San Raffaele Scientific Institute and the Università Vita-Salute San Raffaele, Milan, Italy (A.A.M.); Heart Transplantation Division, Ospedale Niguarda Ca' Granda, Milan, Italy (E.A.); Centro Cardiologico Monzino, Istituto Di Ricovero e Cura a Carattere Scientifico (IRCCS)Milan, Italy, Department of Cardiovascular Sciences, University of MilanMilan, Italy (M.D.M.); Center for the Study of Atherosclerosis, Italian Society for the Study of Atherosclerosis Lombardia Chapter, Bassini Hospital Cinisello BalsamoMilan, Italy (L.G., F.P., K.G., S.T., G.D.N.); Department of Pharmacological Sciences, Università degli Studi di Milano, Milan, Italy (A.L.C., G.D.N.); Multimedica IRCCS, S.S. Giovanni, Milan (L.G., A.L.C.); Heart Care Foundation, Florence, Italy (E.A., A.M.)

**Keywords:** atherosclerosis, C-c chemokine receptor type 7, chemokines, coronary artery disease, effector memory T cells

## Abstract

**Background—:**

Adaptive T-cell response is promoted during atherogenesis and results in the differentiation of naïve CD4^+^T cells to effector and/or memory cells of specialized T-cell subsets. Aim of this work was to investigate the relationship between circulating CD4^+^T-cell subsets and atherosclerosis.

**Methods and Results—:**

We analyzed 57 subsets of circulating CD4^+^T cells by 10-parameter/8-color polychromatic flow cytometry (markers: CD3/CD4/CD45RO/CD45RA/CCR7/CCR5/CXCR3/HLA-DR) in peripheral blood from 313 subjects derived from 2 independent cohorts. In the first cohort of subjects from a free-living population (*n*=183), effector memory T cells (T_EM_: CD3^+^CD4^+^CD45RA^−^CD45RO^+^CCR7^−^ cells) were strongly related with intima-media thickness of the common carotid artery, even after adjustment for age (*r*=0.27; *P*<0.001). Of note, a significant correlation between T_EM_ and low-density lipoproteins was observed. In the second cohort (*n*=130), T_EM_ levels were significantly increased in patients with chronic stable angina or acute myocardial infarction compared with controls. HLA-DR^+^T_EM_ were the T_EM_ subpopulation with the strongest association with the atherosclerotic process (*r*=0.37; *P*<0.01). Finally, in animal models of atherosclerosis, T_EM_ (identified as CD4^+^CD44^+^CD62L^−^) were significantly increased in low-density lipoprotein receptor and apolipoprotein E deficient mice compared with controls and were correlated with the extent of atherosclerotic lesions in the aortic root (*r*=0.56; *P*<0.01).

**Conclusions—:**

Circulating T_EM_ cells are associated with increased atherosclerosis and coronary artery disease in humans and in animal models and could represent a key CD4^+^T-cell subset related to the atherosclerotic process. **(*J Am Heart Assoc*. 2012;1:27-41.)**

## Introduction

T cells play a key role in the immune response observed during atherogenesis.^1,2^ In the arterial wall, cholesterol accumulation followed by vascular inflammation promotes adaptive T-cell response,^3^ and results in the differentiation of naïve CD4^+^T cells to effector and/or memory cells of specialized T-cell subsets in secondary lymphoid organs and in the chemokine-driven recruitment of specific T-cell and monocyte subsets into the atherosclerotic plaque.^4–7^

Editorial on p 3

Most of T cells found in human atherosclerotic lesions are activated effector and/or memory CD4^+^ that predominate over CD8^+^T cells,^8–10^ with a specific antigenic response.^6,11,12^ The activation of inflammatory pathways in atherosclerotic lesions is reflected also in neutrophils, monocytes, and T lymphocytes present in peripheral blood,^13–16^ supporting the concept of a systemic process with local infiltration in the arterial wall. In animal models, a CCR7-dependent recirculation of T cells and monocytes between secondary lymphoid organs and inflamed tissues and egression from atherosclerotic plaques to blood was observed;^17–19^ suggesting a dynamic process that involves local factors (ie, oxidative and shear stress) and systemic factors (ie, traditional cardiovascular risk factors, activation of platelets, and coagulation system).^20,21^ Of note, inhibition of chemokine receptors that are involved in the recruitment of circulating T cells into the plaque such as CCR5 and CXCR3,^22,23^ attenuates atherosclerotic lesion formation in animal models.^24–27^

In spite of the strong experimental evidence supporting a role for T cells in murine atherosclerosis, their clinical significance in humans remains limited.^28^ Therefore, the characterization of circulating CD3^+^CD4^+^T-cell subsets in patients with different stages of atherosclerosis and with various manifestations of coronary artery disease (CAD) could contribute to clarify the role of these cells on atherosclerotic plaque formation and progression. In humans, forefront technologies such as polychromatic flow cytometry, revealed the presence of hundreds of phenotypically distinct leukocytes in the peripheral blood with specialized profiles and programmed functions that may reflect specific activation in the immune response, even in the absence of an overall alteration in total T-cell number.^29^

Adaptive immune response during atherogenesis involves the expansion of memory T cells, antigen-experienced T cells, which (1) elicit stronger and more sustained immune response upon antigen reexposure and (2) support the cytotoxic function of CD8^+^T cells. Because antigens associated with cholesterol-rich lipoproteins promote adaptive immune response and atherosclerosis in animal models,^5^ we hypothesized that in subjects with atherosclerotic disease, alterations in the levels of circulating T-cell subsets that occur, can be appreciated in peripheral blood.

**Figure 1.**
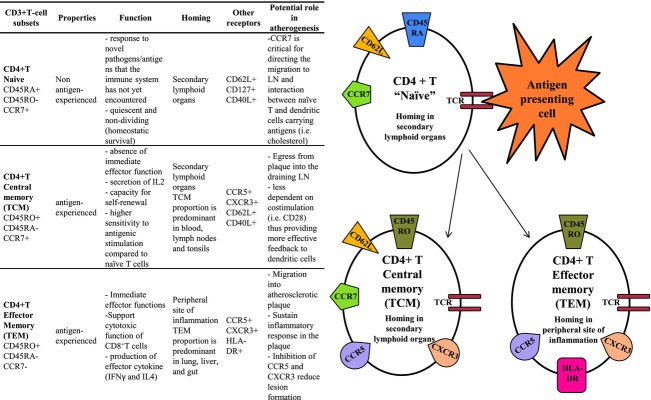
CD4+T cells subsets: properties and potential role in atherosclerosis.

During atherogenesis, antigens generated directly or indirectly as a consequence of hypercholesterolemia and presented by antigen-presenting cells,^7^ are likely to stimulate the development of central memory T cells (T_CM_) and effector memory T cells (T_EM_), from naïve (T_N_) cells. T_N_, T_CM_, and T_EM_ have specific homing capacities and effector functions, and are identifiable by different expression of surface receptors (see Figure 1).^7,30^ Through their expression of CCR7 and CD62L, T_N_ and T_CM_ preferentially home to T-cell areas of secondary lymphoid organs and display little immediate effector functions.^7,30^ On the other hand, T_EM_ which have lost the constitutive expression of CCR7, express tissue homing receptors associated with inflammation (ie, CCR5 and CXCR3) and display more readily effector functions.^7,30^

Aim of this study was to investigate the relationships between atherosclerosis and 7 main CD4^+^T-cell subsets with 50 derived T-cell subpopulations, resulting from the expression of specific markers (CD3/CD4/CD45RO/CD45RACCR7/ CCR5/CXCR3/HLA-DR) investigated by 10-parameter/8-color polychromatic flow cytometry. We first investigated the association between levels of specific T-cell subsets with a surrogate marker of atherosclerosis, that is, the intima-media thickness (IMT) of the common carotid artery in a free-living population. Next, we examined the levels of specific T-cell subsets in patients with stable stenotic coronary atherosclerosis (ie, chronic stable angina [CSA]) and acute unstable manifestations (ie, acute myocardial infarction [AMI]). These analyses identified circulating T_EM_ as the best T-cell subset associated with atherosclerotic disease characterized by a significant correlation with lipid profile. We sought to confirm these findings in animal models by evaluating the correlation of T_EM_ levels with atherosclerosis extent in the aortic root of apolipoprotein E and low-density lipoprotein (LDL) receptor deficient animals.

## Methods

### Study Populations

Institutional Ethics Committees approved the study and informed written consent was obtained from all participating subjects. The study was conducted according to the standards of the Declaration of Helsinki and Good Clinical Practice. Blood samples from 313 subjects from two independent cohort were analyzed.

Carotid study: This cohort was composed by 183 consecutively enrolled subjects from a free-living population during 3 months (September to November 2008) in the context of a larger observational study named PLIC (Progressione Lesioni Intimali Carotidee), previously described.^31^ The clinical and anthropometrical characteristics of this cohort are presented in Table 1.

**Table 1.** Clinical Characteristics and Biological Parameters of Subjects From the General Population (PLIC Study)

	Carotid Study (all subjects)
No.	183

Age (y)	54±14

Male Sex—no. (%)	93 (50.5)

Current smoker no. (%)	43 (23.4)

Hypertension no. (%)	45 (24.8)

Diabetes mellitus no. (%)	14 (7.6)

Hypercholesterolemia no. (%)	31 (16.8)

Systolic blood pressure (mm Hg)	124±18

Diastolic blood pressure (mm Hg)	76±9

BMI (kg/m^2^)	25.9±3.7

Glicemia (mg/dL)	100±22

Total cholesterol (mg/dL)	217±42

LDL-cholesterol (mg/dL)	142±38

HDL-cholesterol (mg/dL)	56±13

Tryglicerides (mg/dL)	100±55

Creatinine (mg/dL)	0.88±0.17

IMT (mm)	0.67±0.14

WBC (10^9^/L)	6.6±1.2

Lymphocytes (10^9^/L)	2.3±0.4

Data are presented as mean±standard deviation. BMI indicates body mass index; HDL, high-density lipoprotein; IMT, intima-medial thickness; LDL, low-density lipoprotein; WBC, whole blood count.

For the study in patients with CAD blood samples were obtained from patients on admission to San Raffaele Scientific Institute and Centro Cardiologico Monzino, both in Milan from December 2007 to June 2009. The control group was made of 40 control subjects age and sex matched without clinical and electrocardiographic signs of CAD. Ninety patients with CAD were divided in a CSA group including 30 patients with effort angina (lasting more than 3 months and without previous history of unstable angina or AMI) and angiographic evidence of coronary artery stenosis (>50%) and an AMI group including 60 patients. AMI samples were obtained before coronary angiography and early after the onset of symptoms when the elevation of troponin I were still minimal (median: 0.4 ng/mL), thus minimizing the possible confounding effect of myocardial necrosis.^32^ A second sample was also obtained from 10 AMI patients after 24 hours from the acute event to check for variation of T-cell subsets during the acute phase. The analysis showed similar levels of these cell subsets (data not shown) suggesting that, at least in the early hours from the onset of AMI, perturbations attributable at myocardial necrosis were limited in the T-cell subsets under investigation. Clinical characteristics and biological parameters of patients with CAD and control subjects are reported in Table 2.

**Table 2.** Clinical Characteristics and Biological Parameters of Patients with coronary artery disease (CAD) and Matched Controls

	Controls	CSA	AMI	*P-*Value
No.	40	30	60	

Age(y)	61 (±9)	64 (±11)	64 (±11)	0.37

Male Sex—no. (%)	32 (80)	22 (73)	46 (77)	0.81

Family history of CAD	17 (43)	12 (40)	20 (33)	0.95

Current smoker	14 (35)	7 (23)	25 (42)	0.23

Hypertension	20 (50)	22 (73)	36 (60)	0.14

Diabetes mellitus	0 (0)	3 (10)	5 (8)	0.14

Hypercholesterolemia	22 (55)	19 (63)	29 (48)	0.40

Troponin I (ng/mL)	–	–	0.4 (0.1–1.1)	–

WBC (10^9^/L)	6.5±1.4	7.9±1.4	11.1±4.0*	<0.0001

Lymphocytes (10^9^/L)	2.2±0.5	2.2±0.4	2.4±0.6	0.11

Monovessel disease	–	11 (37)	22 (37)	0.42

Bivessel disease	–	7 (23)	21 (35)	

Trivessel disease	–	12 (40)	17 (28)	

Aspirin	2 (5)	22 (73)*	36 (60)*	<0.0001

Thienopyridine	0 (0)	8 (27)†	14 (23)†	0.003

β-blockers	1 (3)	14 (47)*	16 (40)*	<0.0001

Statins	1 (3)	14 (47)*	10 (17)†	<0.0001

Ca-blockers	4 (10)	8 (27)	5 (8)	0.04

ACE inhibitors/ARBs	9 (23)	12 (40)	23 (38)	0.19

Nitrates	0 (0)	11 (37)*	24 (40)*	<0.0001

Diuretics	5 (13)	4 (13)	6 (10)	0.87

* <0.001 vs controls after Bonferroni post hoc test.

† <0.01 vs controls after Bonferroni post hoc test.

ACE indicates angiotensin converting enzyme; ARBs, angiotensin receptor blockers; CAD, coronary artery disease; WBC, whole blood count.

### Biochemical Parameters and IMT Measurement

Measurement of biochemical parameters and clinical outcome in the patients of the PLIC study has been described elsewhere.^31,33^ For IMT, briefly, ultrasound scanning and reading of carotid arteries were performed by a single expert sonographer, using an 8-MHz transducer (Biosound 2000 II sa, Indianapolis, IN) with an axial and lateral resolution of 0.385 and 0.500 mm, respectively. The sonographer was blinded to the subject's identity. B-mode evaluations are obtained from captures of the far wall in the first centimeter of common carotid arteries, proximal to the bulb dilation, in lateral projection. Five standardized points 5, 10, 20, 25, and 30 mm from bulb were measured in both arteries and averaged to calculate the mean IMT (IMTm) for each subject. In two scans performed on 75 subjects by the same operator, the mean difference in IMTm was 0.005±0.002 mm and the variation coefficient equal to 1.93%. The correlation between two scans was significant with *r*=0.96 (*P*<0.0001).

### Polychromatic Flow Cytometry

**Figure 2.**
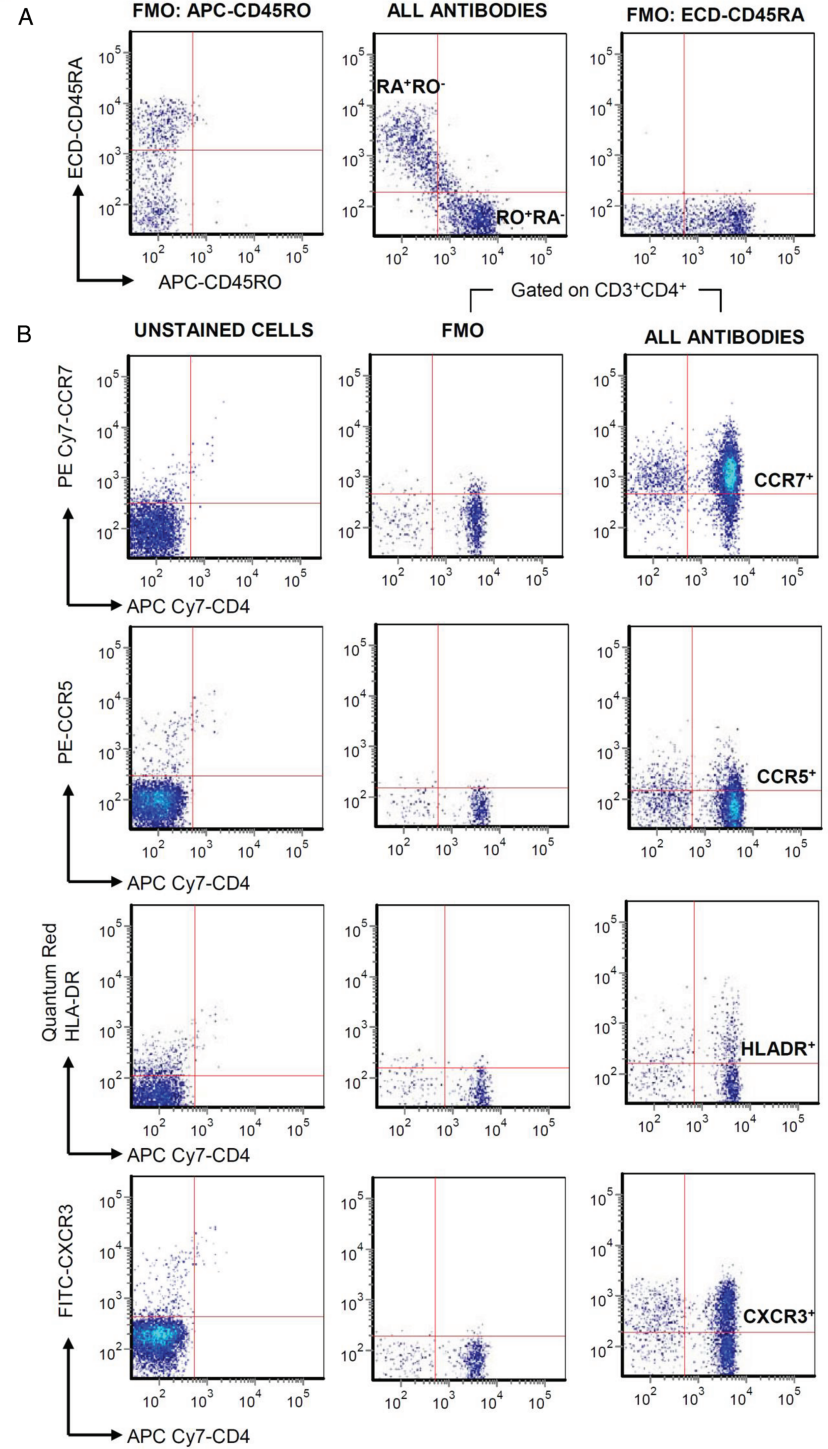
Identification of positive stained cells using the Fluorescence Minus One (FMO) strategy. To appropriately identify positive stained cells and differentiate them from background autofluorescence for gate inclusion, we used the FMO strategy. The background nonspecific fluorescence is collected in the detector assigned to the missing antibody in FMO control. FMO controls are samples that include all the conjugated antibodies but one. The channel for the missing conjugated antibody is that of the FMO gating control. FMO controls are important for setting thresholds in cell populations that express a continuous spectrum of numbers of receptors (such as CD45RO, CD45RA, CCR7, CCR5, HLA-DR, and CXCR3) and allows to better define the positive cell population in comparison with unstained cells. In fact, although unstained cell populations are centered close to zero, their width can vary depending on autofluorescence and spillover corrections in different channels. In (A), the plot on the left the antibody CD45RO-APC conjugated is missing and in the plot on the right the antibody CD45RA-ECD conjugated is missing. In this way, it is possible to correctly define the thresholds of CD45RO positive and CD45RO positive cells in the plot in the center. In (B), the same procedure for the other antibodies used in our experiments. The unstained populations are presented to assess the difference with the positive threshold identified with FMO.

Whole blood from each subject was collected in EDTA anticoagulated vacutainer tube. Samples were stained and fixed within the day of collection. We verified that there were no significant differences in the investigated marker levels in samples stained immediately after collection in comparison to samples stained up to a maximum 24-hour time interval postsampling and stored at room temperature. In order to reduce cellular loss and analysis sampling bias in the specimen, the no wash, whole blood lyses technique was used. For each specimen, 50 μL of a mixture of 8 antibodies was added to 100 μL of whole blood followed by a 20-minute incubation in the dark at room temperature. After staining, the red blood cells were lysed and fixed with the Immune-prep System (Beckman Coulter). White cells, diluted in 1 mL total volume were analyzed on a LSR II Flow Cytometer (BD Biosciences) equipped with four lasers and standard optics. The antibodies were selected to minimize spectral overlap. Furthermore to reduce the nonspecific fluorescence background and optimize the fluorescence signal, we used appropriately titred directly conjugated monoclonal antibodies.^34^ The panel used, consisted of the subsequent cellular surface markers: CD3 (Pacific Blue-labeled, clone HIT3a, BD Pharmingen), CD4 (APC-Cy7, SK3, BD Biosciences), CCR5/CD195 (PE, 2D7, BD Pharmingen), CXCR3/CD183 (FITC, 49801, R&D Systems Inc), CCR7/CD197 (PE-Cy7, 3D12, BD Pharmingen), HLA-DR (Quantum Red, HK14, Sigma-Aldrich), CD45RO (APC, UCHL1, Caltag), and CD45RA (ECD, 2H4, IOTest, Beckman Coulter). To appropriately identify positive stained cells and differentiate them from background autofluorescence for gate inclusion, we used the Fluorescence Minus One strategy. The channel for the missing conjugated antibody is that of the Fluorescence Minus One gating control (Figure 2). Fluorescence intensity for each signal measured was standardized using multiple peak Rainbow calibration particles (Code RCP-30-5A, Spherotech) to allow reproducible and comparable median fluorescence intensity throughout the study period, as previously described.^33^ We identified 7 principal T-cell subsets and 50 T-cell subpopulations derived by principal subsets by means of the combination of the surface markers. All data were acquired in FCS format using FACSDiva Software 5.0 (BD Biosciences). Lymphocytes were identified and electronically gated on forward and orthogonal light scatter signals. The fluorescent signals for phenotype analyses were accumulated for the gated lymphocytes. The instrument raw data were stored electronically to a server for archiving and data processing. Data were processed and analyzed using FCS Express V3 Research edition (De Novo Software, Inc; http://www.denovosoftware.com). Cell viability was >99%, assessed using the Molecular Probes Patented LIVE/DEAD Viability (Invitrogen) according to the manufacturer instructions.

### Identification of T-Cell Subsets in Animal Models of Atherosclerosis by Flow Cytometry

T cell subsets were investigated in peripheral blood collected from male mice with apolipoprotein E or LDL receptor deficiency (two animal models of atherosclerosis) and control animals with the same C57Bl6/J background.^35^ The investigation conformed to the European Commission Directive 86/609/EEC and was approved by the local committee (Progetto di Ricerca 2009/3). Briefly, at 8 weeks of age, animals were fed ad libitum with western-type diet (21% fat, 0.15% cholesterol, and 19.5% casein, Harlan, Bresso, Italy) for 16 weeks except for the group 1 (C57Bl6/J animals that continued at chow diet). Mice were euthanized with an overdose of Avertin 2.5% (Aldrich Chemical Co ), followed by cervical dislocation. Blood samples were collected in EDTA tubes immediately before death and plasma was separated by low-speed centrifugation at 4°C. The measurement of plasma lipids was performed by enzymatic techniques (ABX for Cobas Mira Plus, Montpellier, France)^35^ and atherosclerosis was quantified at the aortic sinus as described.^36^ For flow-cytometry analysis, the following panel of cellular surface markers was used: CD4 (Pacific Blue-labelled, RPA-T4, BD Pharmingen), CD44 (FITC, IM7, BD Pharmigen), and CD62L (APC, mMEL-14, BD Pharmigen).

### Monitoring T-Cell Subsets in Subjects Who Underwent Tetanus Vaccination

For this study, blood samples from three control subjects that performed booster shots of tetanus vaccine for personal reasons (travel to a zone at increased risk for tetanus) were taken. The subjects signed an informed consent. Subjects were sampled (4 mL of whole blood in EDTA each time) the day of the vaccination (before exposure), and 2, 7, 14, and 21 days after exposure. All three subjects were previously vaccinated. They received a single dose of 40 International Units of tetanus toxoid (ANATETALL, Novartis Vaccines & Diag) by intramuscular injection.

### Statistical Analysis

Results are reported as mean±standard deviation (SD) or median, first to third interquartile on the basis of normal or nonnormal distribution respectively (Shapiro-Wilk normality test was performed). Group differences in continuous variables were determined by using Student's *t* test or one-way analysis of variance with Bonferroni's multiple comparison test for normally distributed values and Mann-Whitney U test or Kruskall-Wallis test with Dunn's multiple comparison test as appropriate. Analysis of variance univariate analysis was performer between all variables and IMT. Multiple stepwise regression analysis was performer with IMT as the dependent variable, and by entering the independent variable with the highest partial correlation coefficient at each step. Associations are summarize using correlation coefficients, Pearson correlation coefficient is shown for variables normally distributed while Spearman rank correlation coefficient is shown for variables that were not normally distributed. Group differences or correlations with *P*<0.05 were deemed as statistically significant. GraphPad Prism 4 and SPSS Statistics 17.0 softwares were used for analysis.

## Results

### Identification of Seven Main CD3^+^CD4^+^T-Cell Subsets and 50 Derived T-Cell Subpopulations

**Figure 3.**
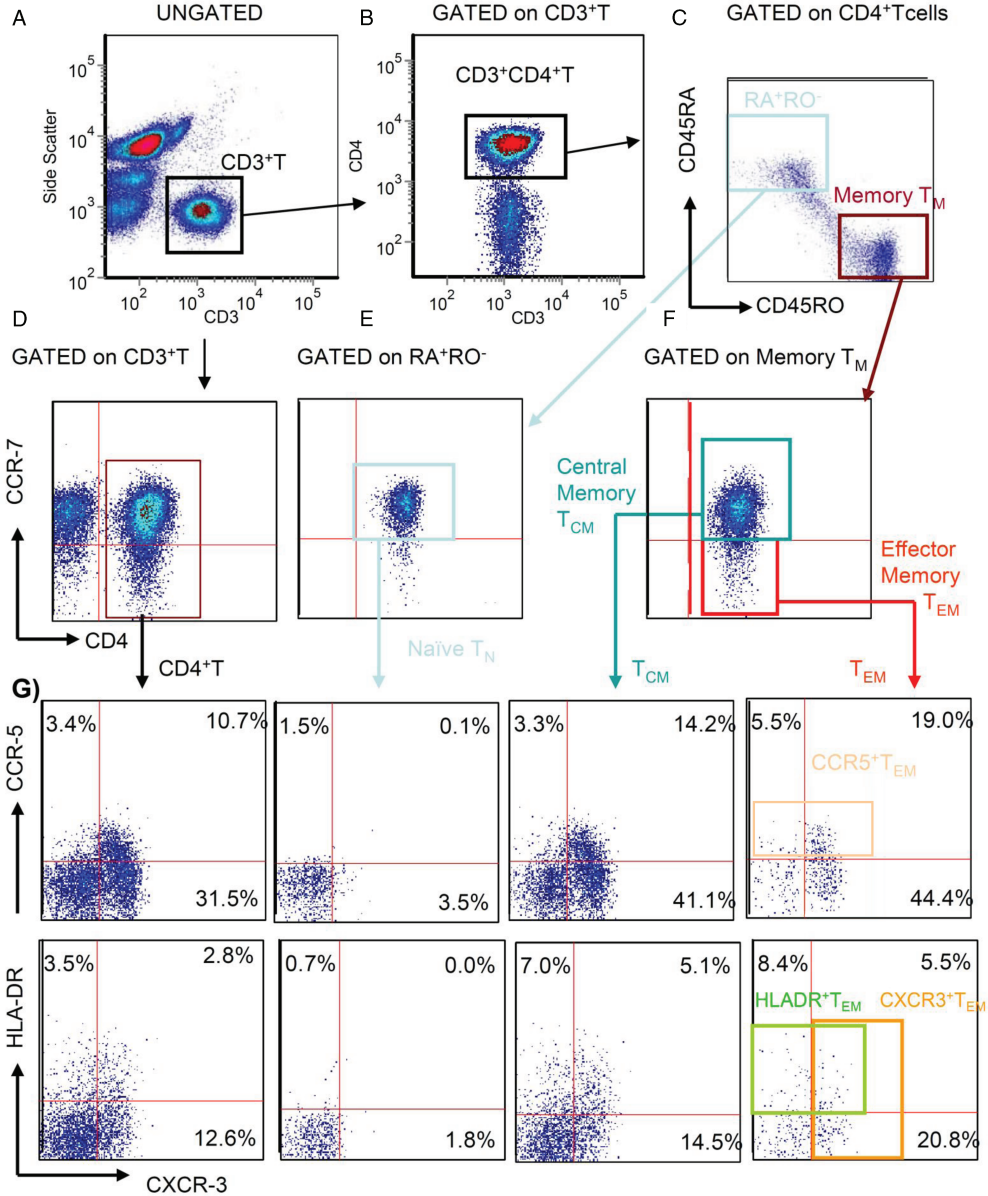
Gating strategy to identify the principal CD4^+^T-cell subsets, and some of the T-cell subpopulations. Color dot plot of a representative subject. Lymphocytes were identified and electronically gated on orthogonal light scatter signals and CD3 immunopositivity (approximately 30.000 events on CD3 for each sample) (A). Then CD3^+^CD4^+^T cells were identified (B). Gating on CD3^+^CD4^+^T cells, memory T cells (T_M_) were identified as CD45RA negative and CD45RO positive (C). In (D), the expression of the chemokine receptor CCR-7 was shown in CD3^+^T cells. In (E), the expression of CCR7 defined the naïve T cells (T_N_) as CD45RA^+^CD45RO^−^ and CCR7 positive. In (F), the expression of CCR7 is used to define central memory T cells (T_CM_; CCR7 positive) and effector memory T cells (T_EM_; CCR7 negative). In (G), it is shown the different expression of chemokine receptors CXCR3, CCR5, and marker of activation HLA-DR in CD3^+^CD4^+^CCR7^+^ T cells, in T_N_, in T_CM_ and in T_EM_. T_EM_ have a relative increased expression of markers of activation and chemokine receptors in comparison with other T-cell subsets. In G are shown some of the T-cell subpopulations that are considered in further analysis (such as CXCR3^+^T_EM_, CCR5^+^T_EM_, HLA-DR^+^T_EM_).

We identified seven principal T-cell subsets by 10-parameter/8-color flow cytometry analysis: naïve T cells (called T_N_ defined as CD3^+^CD4^+^CD45RA^+^ CD45RO^−^CCR7^+^), memory T cells (T_M_, defined as CD3^+^CD4^+^CD45RA^−^CD45RO^+^), central memory T cells (T_CM_, defined as CD3^+^CD4^+^CD45RA^−^CD45RO^+^CCR7^+^) and effector memory T cells (T_EM_, defined as CD3^+^CD4^+^CD45RA^−^CD45RO^+^CCR7^−^), HLA-DR^+^T cells (CD3^+^CD4^+^HLA-DR^+^), CCR5^+^T cells (CD3^+^CD4^+^CCR5^+^), and CXCR3^+^T cells (CD3^+^CD4^+^CXCR3^+^) (Figure 3). Then, we described 50 T-cell subpopulations derived from these principal T cell subsets (see Table 3 for the list of all analyzed subpopulations). Principal T-cell subsets and subpopulations were expressed as percentage of total CD3^+^CD4^+^T cells.

**Table 3.** Description of All T-Cells Subpopulation Analyzed

CD3^+^CD4^+^T Subsets	Subpopulations	Levels (% of CD3^+^CD4^+^)
Naïve CD45RA^+^RO^−^CCR7^+^		

	CXCR3^+^	1.1 (1.1)

	CCR5^+^	0.9 (0.8)

	HLA-DR^+^	0.2 (0.2)

	CXCR3^+^CCR5^+^	>0.1*

	CXCR3^+^CCR5^−^	0.1 (0.1)

	CXCR3^−^CCR5^+^	>0.1*

	CXCR3^+^HLA-DR^+^	>0.1*

	CXCR3^+^HLA-DR^−^	1.0 (1.0)

	CXCR3^−^HLA-DR^+^	>0.1*

CD45RA^+^RO^−^CCR7^−^†		2.4 (1.7)

	CXCR3^+^	0.1 (0.1)

		

Central memory T CD45RA^−^RO^+^CCR7^+^		

	CXCR3^+^	20.1 (7.3)

	CCR5^+^	5.2 (3.6)

	HLA-DR^+^	3.8 (1.9)

	CXCR3^+^CCR5^+^	3.1 (2.4)

	CXCR3^+^CCR5^−^	17.1 (6.1)

	CXCR3^−^CCR5^+^	2.2 (1.7)

	CXCR3^+^HLA-DR^+^	1.8 (1.1)

	CXCR3^+^HLA-DR^−^	18.4 (6.9)

	CXCR3^−^HLA-DR^+^	2.0 (1.2)

Effector memory T CD45RA^−^RO^+^CCR7^−^		

	CXCR3^+^	5.2 (2.5)

	CCR5^+^	0.8 (0.5)

	HLA-DR^+^	1.0 (0.7)

	CXCR3^+^CCR5^+^	2.0 (1.9)

	CXCR3^+^CCR5^−^	3.9 (2.1)

	CXCR3^−^CCR5^+^	0.6 (0.5)

	CXCR3^+^HLA-DR^+^	0.4 (0.3)

	CXCR3^+^HLA-DR^−^	4.2 (2.3)

	CXCR3^−^HLA-DR^+^	0.6 (0.4)

		

CD45RA^+^RO^+^‡		5.6 (3.5-8.5)

CD45RA^+^RO^+^CCR7^+^		5.2 (3.1-7.8)

	CXCR3^+^	1.1 (0.8-1.5)

	CCR5^+^	0.5 (0.3-0.7)

	HLA-DR^+^	0.1 (0.1)

	CXCR3^+^CCR5^+^	0.4 (0.2–0.5)

	CXCR3^+^CCR5^−^	0.7 (0.5–1.1)

	CXCR3^−^CCR5^+^	0.1 (0.04–0.2)

	CXCR3^+^HLA-DR^+^	>0.1*

	CXCR3^+^HLA-DR^−^	1.0 (0.7–1.4)

	CXCR3^+^HLA-DR^+^	>0.1*

CD45RA^+^RO^+^CCR7^−^		0.4 (0.2–0.7)

		

	CXCR3^+^	0.1 (0.04–0.13)

	CCR5^+^	>0.1*

	HLA-DR^+^	>0.1*

	CXCR3^+^CCR5^+^	>0.1*

	CXCR3^+^CCR5^−^	0.1 (0.03–0.11)

	CXCR3^−^CCR5^+^	>0.1*

	CXCR3^+^HLA-DR^+^	>0.1*

	CXCR3^+^HLA-DR^−^	0.1 (0.04–0.11)

	CXCR3^+^HLA-DR^+^	>0.1*

Data are presented as mean±standard deviation or median (25th quartile to 75th quartile) as appropriate.

* We did not report precise values in case of small subpopulations with frequencies below 0.1% as median or mean value.

† CD45RA^+^RO^−^CCR7^−^ is a naive subpopulation that had lost the expression of CCR7. This subpopulation is small and non–well characterized.

‡ CD45RA^+^RO^+^ represents a subpopulation of transition from a naïve to memory phenotype that coexpresses both CD45RA and CD45RO.

### Antigen Reexposure Can Modify the Composition of Memory T-Cell Compartment in Humans

**Figure 4.**
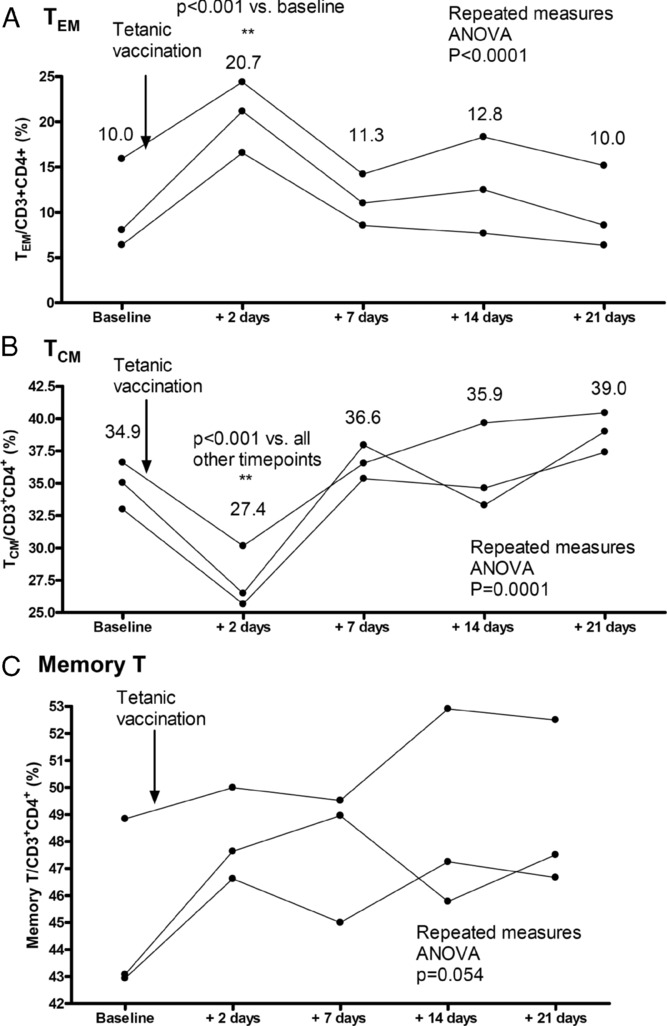
Effector memory T cell (T_EM_), central memory T cell (T_CM_), and memory T cell variations at several time points after tetanic vaccination in three healthy subjects. Dots represent subject and continuous lines show the temporal changes between the first sampling (before the tetanic vaccination) and the following ones at several time point after the antigen exposure (after 2, 7, 14, and 21 days). We observed a temporally limited increase in levels of T_EM_ and a reciprocal decrease in levels of T_CM_ at 2 days after antigen exposure with a subsequent decrease of T_EM_ and increase of T_CM_ to prevaccination levels after 1 and 3 weeks after the exposure. Means percentage are reported and referred to the total number of CD3^+^CD4^+^T cells (A, B). A trend vs a significant increase in total memory T cells have been observed after 2 to 3 weeks from tetanic vaccination (C).

As proof of principle in a limited number of subjects, we analyzed changes in the composition of memory T-cell compartment after antigen reexposure. We demonstrated that our approach was sensitive enough to clearly identify these modifications. In particular, antigen reexposure lead to a temporally limited T_EM_ increase in the peripheral blood of control subjects vaccinated with tetanus toxoid (For details also concerning T_M_ and T_CM_ modifications see Figure 4).

### Correlations Between Circulating T-Cell Subsets and Cardiovascular Risk Factors

First, we investigated the clinical variables that can affect the distribution of T-cell subsets under investigation. Overall, men and women had similar CD3^+^CD4^+^T cells (61.9±9.1% versus 59.5±11.5%, *P*=0.13; expressed as percentage of total CD3^+^ lymphocytes). Male subjects had lower T_N_ (30.0±12.1% versus 34.6±12.9%, *P*=0.01; percentage of CD3^+^CD4^+^T cells), but higher T memory cells (62.7±12.1% versus 57.8±14.4%, *P*=0.01) including higher T_EM_ (11.9±4.9% versus 10.3±3.7%, *P*=0.01) and T_CM_ (50.9±10.1% versus 47.6±12.3%, *P*=0.04) compared with female (Table 4). T_N_ were inversely correlated with age (Spearman *r*=−0.31, *P*<0.01), while T_M_ (Spearman *r*=0.32, *P*<0.01) including T_EM_ (Spearman *r*=0.31, *P*<0.01) and T_CM_ (Spearman *r*=0.22, *P*<0.01) or HLA-DR^+^T cells (Spearman *r*=0.20, *P*<0.01) were directly correlated with age.^37,38^

**Table 4.** T-Cell Principal Subpopulation Distribution According to the Gender

T lymphocytes	n183	♂ 93	♀ 91
CD3+CD4+			

CCR7+ (%)	85.8±4.8	85.1±5.5	86.6±4.1

CCR5+ (%)	6.9±4.1	7.3±4.4	6.6±3.8

CXCR3+ (%)	26.4±9.1	27.4±10.0	25.5±8.0

HLA-DR+ (%)	4.9±2.4	5.0±2.	4.8±2.7

Naïve CD45RA+RO- CCR7+ (%)	32.3±12.7	29.9±12.1	34.6±12.9

Memory CD45RA-RO+(%)	60.3±13.4	62.8±12.1	57.8±14.4

Central memory (%)	49.3±11.4	50.9±10.1	47.6±12.3

Effector memory (%)	11.1±4.4	11.9±4.9	10.3±3.7

Data are presented as mean±standard deviation.

Hypercholesterolemic subjects showed increased levels of T_EM_ (mean±SD 12.9±1.0% versus 10.7±0.3%, *P*=0.02) and HLA-DR^+^ T cells (6.2±0.7% versus 4.6±0.2%, *P*=0.03). The presence of hypertension was associated with decreased levels of T_N_ (27.2±1.9% versus 33.6±1.4%, *P*<0.01) and increased levels of T_M_ (66.3±2.0% versus 58.7±1.2%, *P*<0.01), including T_CM_ (53.5±1.6% versus 43.2±1.4%, *P*=0.01) and T_EM_ (12.9±0.8% versus 10.6±0.4%, *P*<0.01). Smoking habits were not associated with increased TEM levels (data not shown).

Different correlations between T-cell subsets and cardiometabolic risk factors were observed. Upon age-adjustment, T_EM_ were significantly and directly correlated with plasma total cholesterol, triglycerides, LDL cholesterol, glycemia, body mass index, and systolic blood pressure. Furthermore, subjects with metabolic syndrome or diabetes had significantly higher levels of T_EM_ (14.1±4.8% and 13.8±5.1%, respectively) compared with subjects without metabolic syndrome (10.8±1.2%, *P*=0.02) or without diabetes (10.8±1.3%, *P*=0.03). No significant correlation was observed between T_CM_ and cardiometabolic risk factors, while T_N_ were inversely correlated only with plasma triglyceride levels. Among other main subsets analyzed, HLA-DR^+^ T cells showed a direct correlation with total cholesterol, triglycerides, and LDL cholesterol, and an inverse correlation with HDL cholesterol.^39^

### Correlations Between Circulating T-Cell Subsets and Carotid IMT

Second, we investigated whether specific T-cell subsets are associated with preclinical atherosclerosis, defined by IMT at the common carotid levels. Table 5 summarizes the correlations between IMT with clinical, lipid, and metabolic variables, and T-cell subsets. IMT correlated positively with age, systolic blood pressure, total, and LDL cholesterol, triglyceride levels, body mass index, and glycemia, whereas IMT inversely correlated with high-density lipoprotein cholesterol levels. As expected, several correlations were lost after age-adjustment of the IMT (Table 5). The analysis of CD3^+^CD4^+^T-cell subsets revealed that T_M_, T_EM_, and HLA-DR^+^T cells significantly correlate with IMT, whereas T_N_ inversely correlates with IMT. As age is the known main determinant of carotid IMT, and several CD3^+^CD4^+^T-cell subsets and cardio-metabolic risk factors correlated with age, the analysis of the correlation between T-cell subsets and IMT was performed after age-adjustment. A significant direct correlation remained between age-adjusted IMT and T_EM_ (*P*<0.001), whereas only a trend for correlation was observed between age-adjusted IMT and HLA-DR^+^ (*P*=0.07).

**Table 5.** Unadjusted and Age-Adjusted Correlation Between Intima-media thickness (IMT) and Clinical, Lipid, Metabolic and Inflammatory Parameters, and T-Cell Subsets (Pearson Correlation Coefficients are Shown, *n* = 183)

IMT			Age-adjusted IMT	

	*r*	*P*	*r*	*P*
Age	0.63	<0.001	-	-

Clinical variables				

Systolic blood pressure	0.33	<0.001	0.07	0.38

Diastolic blood pressure	0.12	0.14	−0.04	0.61

Lipid profile				

Total cholesterol	0.15	0.04	−0.11	0.12

LDL cholesterol	0.19	0.01	−0.07	0.33

HDL cholesterol	−0.26	0.001	−0.23	0.03

Triglyceridemia	0.25	0.001	0.06	0.42

Metabolic profile				

Body mass index	0.26	<0.001	0.07	0.36

Creatinine	0.34	<0.001	0.15	0.05

Glycemia	0.27	<0.001	0.14	0.07

Inflammatory profile				

CRP	0.08	0.43	−0.02	0.84

T-lymphocytes profile				

CD3^+^CD4^+^	−0.08	0.28	−0.11	0.11

Naïve CD45RA^+^RO^-^CCR7^+^	−0.19	0.01	0.10	0.18

Memory CD45RA^-^RO^+^	0.18	0.02	−0.05	0.54

Central memory T	0.06	0.42	−0.10	0.20

Effector memory T	0.40	<0.001	0.27	<0.001

CCR5^+^	0.08	0.30	0.10	0.18

CXCR3^+^	0.14	0.05	0.04	0.63

HLA-DR^+^	0.28	<0.001	0.14	0.07

LDL indicates low-density lipoprotein; HDL, high-density lipoprotein; CRP, C-reactive protein.

In the multiple regression analysis, age, creatinine, and T_EM_ cells emerged as independent predictors of IMT (Table 6). We cannot exclude that the relatively limited number of subjects studied could have underestimated the impact of the lipid profile on the IMT.

**Table 6.** Overall Multiple Regression Analysis Between Intima-Media Thickness (IMT; dependent variable) and Age, Gender, Smoking Habits, Clinical Parameters, and T-Cell Subsets that Correlate with IMT in the Single Model

	Model	
Independent variables	β	*P*
Age	0.56	<0.01

Gender	0.05	0.67

Smoking habits	−0.03	0.98

Systolic Blood Pressure	−0.02	0.82

Total cholesterol	−0.12	0.13

LDL cholesterol	−0.11	0.11

HDL cholesterol	−0.10	0.09

Triglyceridemia	0.01	0.89

Body mass index	−0.05	0.51

Creatinine	0.19	<0.01

Glycemia	0.05	0.55

CD3+CD4+		

Naive T cells	−0.12	0.19

Central memory T cells	0.11	0.56

Effector memory T cells	0.22	<0.01

CCR5+	−0.07	0.34

CXCR3+	−0.06	0.45

HLA-DR+	0.09	0.26

BMI indicates body mass index; HDL, high-density lipoprotein; LDL, low-density lipoprotein.

### T-Cells Subset Levels in Controls and Patients With CAD

**Figure 5.**
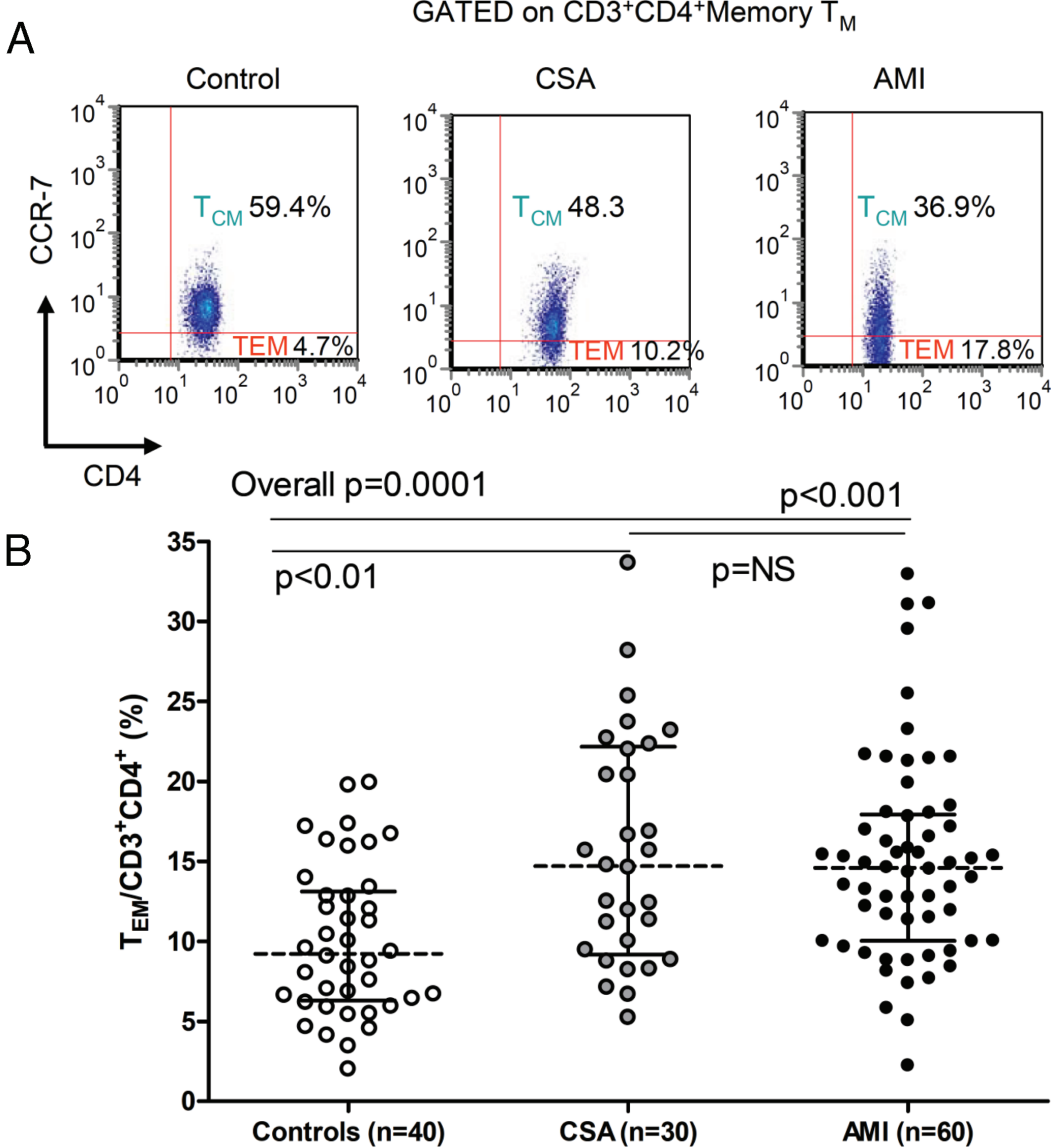
Effector memory T cells (T_EM_) levels are increased in patients with coronary artery disease (CAD). Representative color dot plots from a control and patients with different CAD manifestations: CSA, chronic stable angina; AMI, acute myocardial infarction (A). Significant increases in T_EM_ levels were observed in patients with CSA and AMI in comparison with controls. There were no significant difference in levels of T_EM_ between CSA and AMI. Kruskall-Wallis and Dunn's test were performed. Dots represent individual patient data; dashed lines show median value and continuous lines show 25th and 75th percentiles (B).

To further confirm the association of T_EM_ with the extent of atherosclerosis, we studied the levels of T_EM_ in relation to symptomatic atherosclerosis in the coronary district. Levels of CD3^+^CD4^+^T-cell subsets were investigated in a second independent cohort of patients with stable and unstable manifestations of CAD compared with age- and sex-matched controls. While T_N_ and T_CM_ levels were not different among patients with CSA, AMI, and controls; T_EM_ resulted in significant increase in both CSA and AMI patients compared with controls (*P*<0.01 for both) with similar levels between CSA and AMI patients (Figure 5 and Table 7). Similarly, HLA-DR^+^T cells were significantly increased in patients with CSA and AMI compared with controls (*P*<0.01 for both), without differences between CSA and AMI patients. CCR5^+^T cells and CXCR3^+^T cell levels did not differ among controls, and patients with CSA and AMI (Table 7).

**Table 7.** Circulating T-Cell Subset Levels in Controls, Patients With Chronic Stable Angina (CSA) and Acute Myocardial Infarction (AMI)

	Controls (*n* = 40)	CSA (*n* = 30)	AMI (*n* = 60)
T-lymphocytes profile CD3^+^CD4^+^			

*Naïve CD45RA^+^RO^-^CCR7^+^*	29.3 (18.3–35.2)	24.0 (17.9–33.8)	24.4 (19.7–32.8)

*Memory CD45RA^-^RO^+^*	59.5 (15.9)	65.0 (12.4)	62.8 (12.6)

Central memory T	49.3 (14.5)	48.9 (10.9)	46.6 (11.7)

Effector memory T	9.2 (6.3–13.1)	14.7 (9.2–22.2)*	14.6 (10.0–17.9)†

CCR5^+^	9.1 (5.9–12.3)	7.0 (5.2–17.4)	9.3 (5.4–18.3)

CXCR3^+^	27.5 (22.1–33.0)	28.9 (23.7–35.4)	28.2 (21.3–31.5)

HLA-DR^+^	2.2 (1.3–4.0)	5.8 (3.7–8.4)†	5.9 (4.0–9.9)†

* <0.01

† <0.001 vs controls.

Overall *P*=0.0001 for Effector Memory T (T_EM_), and overall *P*=0.002 for HLA-DR^+^ (Kruskal-Wallis test). Results are expressed as percentage of total CD3^+^CD4^+^T cells. Values are presented as mean (standard deviation) or as median (1° to 3° quartile) as appropriate.

### Circulating T_EM_-Cell Subpopulations in Subclinical Atherosclerosis and in CAD

**Figure 6.**
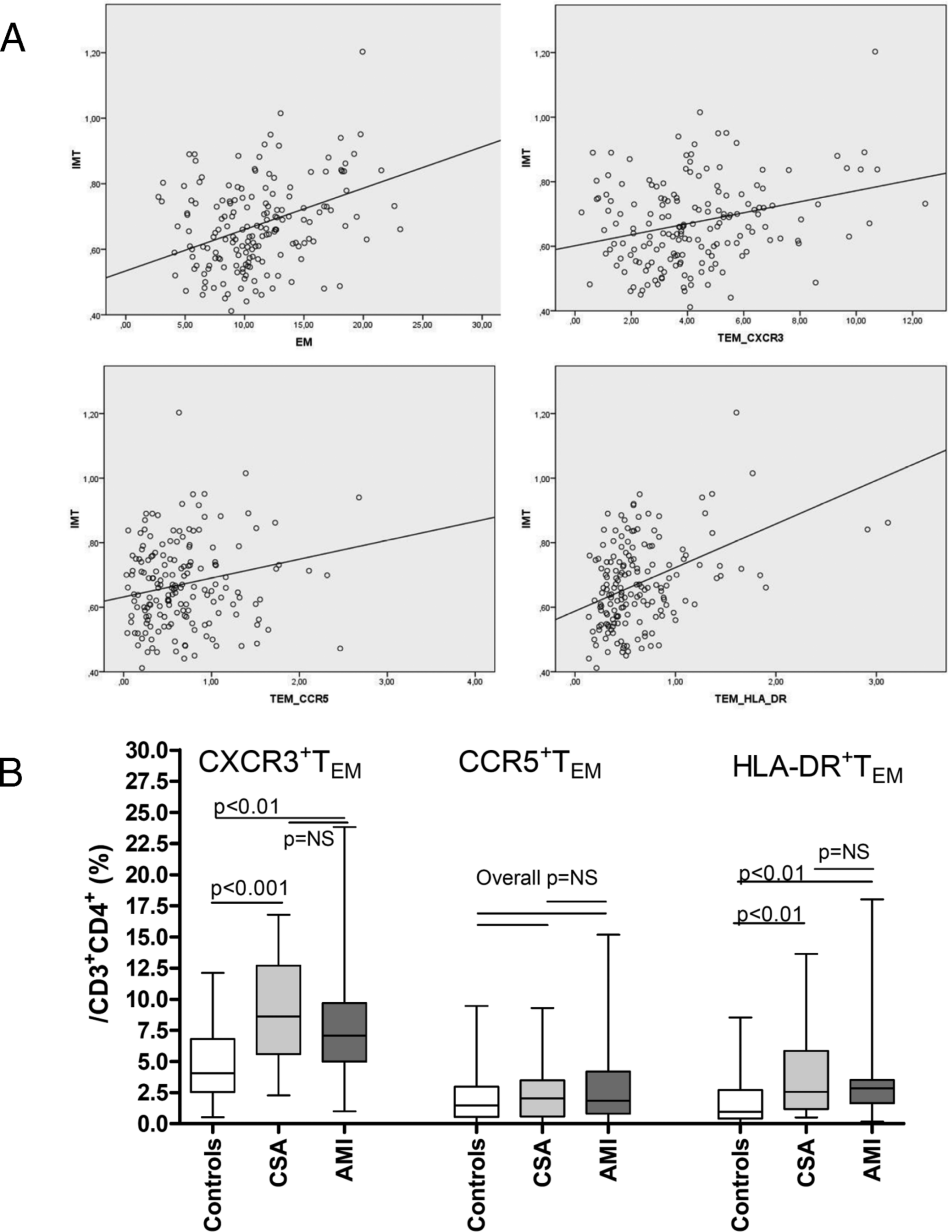
Effector memory T-cell (T_EM_) subpopulations correlate with intima-media thickness (IMT) and are increased in patients with coronary artery disease (CAD). The correlation between IMT and T_EM_, CXCR3^+^T_EM_, HLA-DR^+^T_EM_, and CCR5^+^T_EM_ is shown in A to D (see Methods Section for details). (E) The percentage of CXCR3^+^T_EM_, HLA-DR^+^T_EM_, and CCR5^+^T_EM_ in patients with chronic stable angina (CSA, *n* = 30) or acute myocardial infarction (AMI, *n* = 60) and controls (*n* = 40). The Kruskal-Walli test with Dunn's comparison for all groups was used.

Given the potential association between T_EM_ and atherosclerosis and to further investigate whether any of the T_EM_ subpopulations could better explain this finding, the correlation between CXCR3^+^T_EM_, CCR5^+^T_EM_, HLA-DR^+^T_EM_ subpopulations, and IMT was investigated. Compared with all T_EM_ that showed a strong correlation with IMT (Spearman *r*=0.27, *P*<0.01), HLA-DR^+^T_EM_ showed the highest degree of correlation (Spearman *r*=0.37, *P*<0.01), with CCR5^+^T_EM_ showing a degree of correlation similar to T_EM_ (Spearman *r*=0.25, *P*<0.01) while a lower but still significant correlation was observed also for T_EM_ CXCR3^+^T_EM_ and IMT (Spearman *r*=0.15, *P*<0.05) (Figure 6A). HLA-DR^+^T_EM_ and CXCR3^+^T_EM_ resulted the independent covariates associated with IMT (Table 8). These data were supported with the findings in patients with CAD. Indeed HLA-DR^+^T_EM_ subpopulation resulted significantly increased in both CSA and AMI patients compared with controls (*P*<0.01 for both) and, again, HLA-DR^+^T_EM_ levels were not different in CSA and AMI. Also CXCR3^+^T_EM_ were significantly increased in CSA and AMI patients compared with controls (overall *P*=0.002), whereas no differences were observed for CCR5^+^T_EM_ (Figure 6B). These data suggest that a specific T_EM_ subpopulation, namely HLA-DR^+^T_EM_, which express the activation marker HLA-DR, could better reflect the association with the atherogenic process.

**Table 8.** Relative Contribution of Each Effector Memory T-Cell Subset to IMT Prediction. Data From Forced Entry Multiple Regression Analysis With IMT as Dependent Variable and T_EM_, T_EM_-HLA-DR+, T_EM_-CCR5+, and T_EM_-CXCR3+ as Covariates Are Shown

Model		Standardized Coefficient	*t*	Significance	Confidence Intervals 95.0% for B	

	Beta				Lower limit	Upper limit
1	(Constant)		21.554	0.000	0.528	0.634

	EM	−0.227	−1.333	0.184	−0.018	0.003

	TEM_CCR5	−0.028	−0.381	0.704	−0.048	0.032

	TEM_CXCR3	0.370	2.569	0.011	0.005	0.038

	TEM_HLA_DR	0.432	4.425	<0.001	0.072	0.189

### T_EM_ Cells Were Increased in Animal Model of Atherosclerosis and Correlate With the Extent of Atherosclerosis

**Figure 7.**
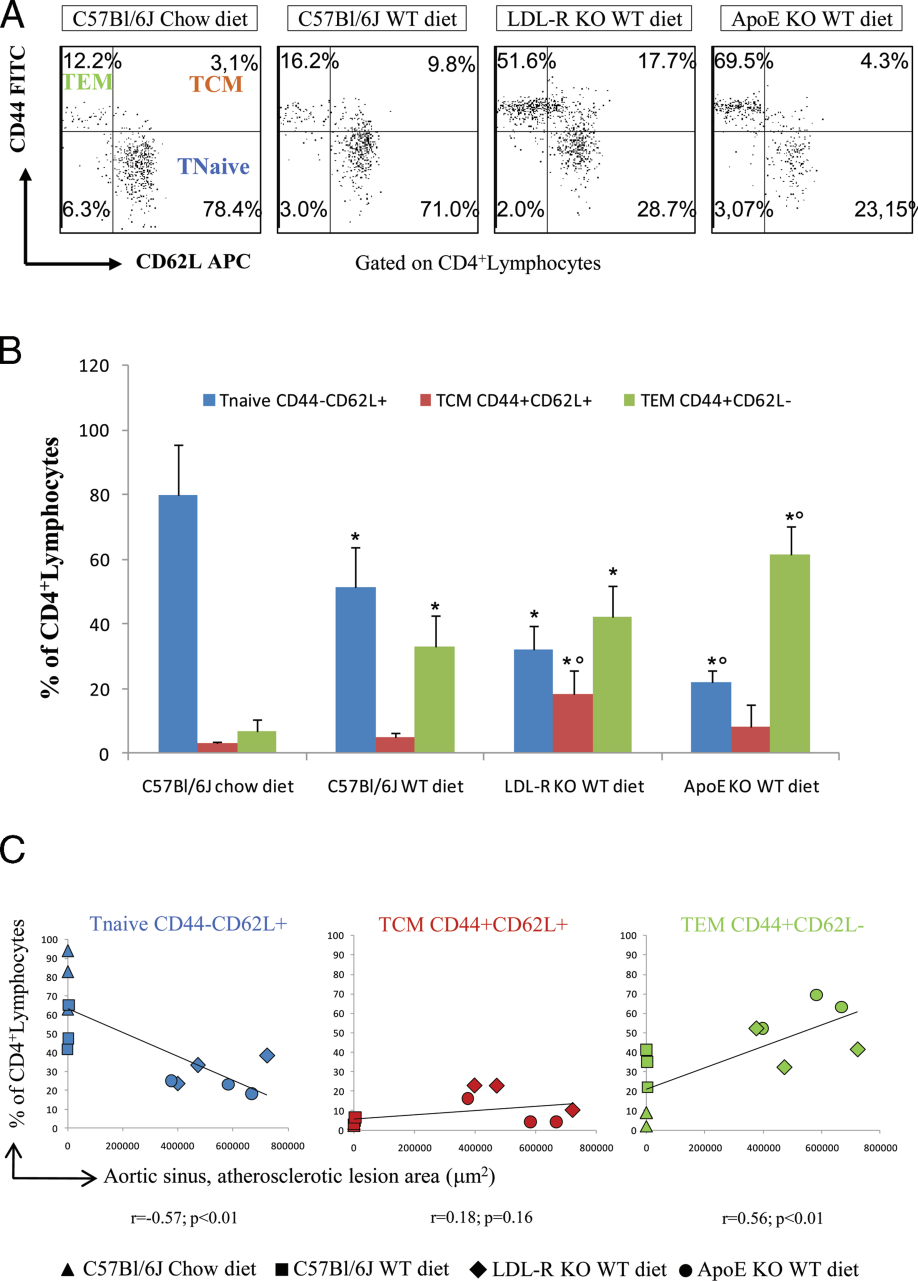
Effector memory (T_EM_) levels are increased in animal models of atherosclerosis. Representative color dot plots from wild-type (C57BL/6J) animals fed a chow diet, a cholesterol rich (western type, WT) diet and from LDL receptor (LDL-R), and apolipoprotein E (Apo-E) knockout animals are shown in (A), while the percentages of circulating naïve T cells (T_N_: in mouse defined as CD62L^+^CD44^−^), central memory T cells (T_CM_: CD62L^+^CD44^+^), and memory effector T cells (T_EM_: CD62L^−^CD44^−^) are shown in (B) (*n* = 12; 3 for each group) (*P<0.05 vs C57Bl/6J chow diet; °*P*<0.05 vs C57Bl/6J WT diet, pairwise test with Bonferroni correction). The correlations between circulating levels of T_N_, T_CM_, and T_EM_ and atherosclerotic plaque area at the aortic sinus are shown in panel C (*n* = 12; 3 for each group, Spearman correlation coefficients are shown).

To further characterize the presence of T_EM,_ T_CM_, or T_N_ cells in relation to the extent of atherosclerosis, the presence of CD4^+^CD44^−^CD62L^+^ (T_N_), CD4^+^CD44^+^CD62L^−^ (T_EM_), and of CD4^+^CD44^+^CD62L^+^ (T_CM_) was investigated in animal models of atherosclerosis fed an atherogenic diet (Figure 7A).^40^ Of note, the percentage of T_N_ significantly decreased while that of T_EM_ significantly increased in LDL receptor knockout and apolipoprotein E knockout animals compared with control animals (Figure 7B). T_CM_ were significantly increased in LDL receptor knockout mice (Figure 7B). Of interest, the percentage of T_N_ was inversely correlated with the extent of atherosclerotic lesions in the aortic sinus of the animals (*r*=−0.57; *P*<0.01) while that of T_EM_ was directly correlated (*r*=0.56; *P*<0.01; Figure 7C), further supporting the link between T_EM_ and atherosclerosis also in animal models.

## Discussion

Several studies have suggested a role of CD4^+^T cells in the pathogenesis of atherosclerosis.^2,5,41^ The present study is, to date, the largest one which investigated circulating CD4^+^T-cell subsets in relation to atherosclerotic disease in humans. The simultaneous assessment of 8 independent membrane T-cell molecules by polychromatic flow cytometry allowed to identify more accurately and in relatively unbiased manner the phenotype of circulating T cells.

T_EM_ emerged as the T-cell subset with the strongest association with atherosclerosis in carotid and coronary vascular districts at different stages of the process. T_EM_ were significantly and directly correlated with plasma total cholesterol and LDL cholesterol, although the association between T_EM_ and carotid atherosclerosis was independent of the classical cardiovascular risk factors, supporting the relevance of adaptive immune response in cardiovascular disorders.^28,41^

Partial characterizations of circulating T cells, which had been previously performed, are consistent with our results: indeed increased levels of CD28^null^ or TCR^dim^T cells and decreased levels of CCR7 have been reported in patients with CAD,^16,27,42–44^ whereas an expansion of the overall memory T-cell population has been associated with the extent of IMT in elderly Japanese subjects.^45^

T_CM_ and T_EM_ cells persist in the memory pool once the antigen that elicited an immune response has been eliminated. They keep memory of (1) antigen specificity, (2) array of cytokines they had produced, and (3) the site where their effector function is needed. Upon antigen reexposure, T_EM_ display immediate effector functions in inflamed peripheral tissues, whereas T_CM_, activated by dendritic cells in secondary lymphoid organs, generate successive waves of effector cells.^7,30^ CD4^+^T_EM_ are normally excluded from resting lymph nodes and migrate in a CD62P-dependent fashion into reactive lymph nodes or to inflamed tissue, mainly by the expression of CCR5 and CXCR3. The expression of HLA-DR constitutes a marker of effector function acquisition.^40^ The function of T cells depends on their ability to exploit integrins, selectins and chemokine receptors to extravasate and migrate to sites where antigen is present. Of note, increased levels of T_EM_ have been previously described in vasculitis and chronic graft versus host disease.^46,47^ Also in these conditions, altered levels of human T_EM_ were not accompanied by significant changes in T_N_ and T_CM_ pools.^46,47^

### Potential Role of T_EM_ in the Atherosclerotic Process

In hypercholesterolemic animal models it has been observed that CCR7 knockout attenuates ath-erosclerotic plaque development.^17^ This finding stresses the relevance of the differentiation process of naïve CD4^+^T cells to effector and/or memory cells of specialized T-cell subsets (T_CM_ and T_EM_), and the CCR7-dependent T-cell and monocyte migration during atherogensis. However, as CCR7 is expressed on several lymphocytes, including cells that express adhesion molecules required for homing to nonlymphoid tissues,^48^ the deficiency of CCR7 could also impair the trafficking of cells other than CD4 lymphocytes during atherosclerosis. Therefore, the identification of a correlation between a specific memory T-cell subset that lost CCR7 receptor (T_EM_) with atherosclerosis and at the same time, the association with LDL cholesterol levels, support the intriguing interaction between LDL cholesterol, expansion of T_EM_, and atherosclerosis.^5^ Indeed, experimental models have shown that CD4^+^T cells recognize epitopes on native ApoB100 protein. Furthermore, blocking T-cell receptor-dependent antigen recognition by these T cells protects against atherosclerosis.^5,49^ Our data demonstrate that animal models where atherosclerosis derives from impaired lipid metabolism and dyslipidemia, have increased levels of T_EM_ compared with controls that are directly associated with the extent of the atherosclerotic lesions. While these results can support the hypothesis of a causal link between LDL cholesterol and T_EM_ increase, further studies are warranted in patients to investigate whether a sustain antigen stimulation, possibly mediated by LDL cholesterol, could explain the expansion of T_EM_. In this context, although smoking is a strong determinant of total leukocytes count and differential leukocytes subsets, smoking habits were not associated with increased T_EM_ levels. This might suggests other mechanisms associated with smoking deleterious effects on atherosclerosis.

Of note, within the T_EM_ subsets, we have identified subpopulations with a strong correlation with the extent of atherosclerosis, such as those expressing HLA-DR, CXCR3, and CCR5. These receptors modulate the recruitment of T cells into the atherosclerotic plaques,^25,26^ and could thus represent an asset to facilitate T_EM_ patrolling of atherosclerotic plaques at distant sites in the organism. We further confirmed previous reports that had identified the expression of HLA-DR as a T-cell subset associated with CAD, and we identified T_EM_ as the CD4^+^T-cell subset in which HLA-DR is specifically increased.^14,50^

## Conclusion

Among CD4^+^Tcell, T_EM_ and related T_EM_ subpopulations are those strongly correlated with the extent of atherosclerosis in carotid and coronary districts. As T_EM_ are antigen-experienced and long-surviving cells that have lost CCR7 and express HLA-DR, CXCR3, and CCR5, this finding strengthens the concept that the understanding of the inflammatory pathogenesis of atherosclerosis requires careful cellular subphenotyping. Furthermore, the presence of increased levels of circulating T_EM_ in humans and the association with the extent of aortic lesions in animal models, suggest the possibility of targeting plaque chemotaxis and/or antigen encountering as emerging antiatherosclerotic strategies on the top of cardiovascular risk factors control.^25,26,49^
